# Power to the people: To what extent has public involvement in applied health research achieved this?

**DOI:** 10.1186/s40900-016-0042-y

**Published:** 2016-08-17

**Authors:** Gill Green

**Affiliations:** School of Health & Human Sciences, University of Essex, Colchester, UK

**Keywords:** Public involvement, Research, Power, Lay knowledge

## Abstract

**Plain English summary:**

Public involvement is required for applied health research funded in the UK. One of the largest funders, the National Institute of Health Research (NIHR), makes it clear that it values the knowledge of patients and the public. As a result, there are now many resources to make sure that the public voice is included in decision-making about research. However, there is concern that the public voice still has limited impact on research decision-making. This article asks to what extent has power shifted from the scientific research community to the public? It looks at how much power and impact patients and members of the public have about research by asking: How do the public contribute to deciding which research areas and which research projects should be funded? How do they influence how the research is carried out? The article argues that there is evidence that the public voice is present in research decision-making. However, there is less evidence of a change in the power dynamic between the scientific research community and the public. The public involved in research are not always equal partners. The scientific research community still has the loudest voice and patients and the public do not always feel sufficiently empowered to challenge it.

**Abstract:**

Public involvement in applied health research is a pre-requisite for funding from many funding bodies. In particular the National Institute of Health Research (NIHR) in the UK, clearly states that it values lay knowledge and there is an expectation that members of the public will participate as research partners in research. As a result a large public involvement infrastructure has emerged to facilitate this. However, there is concern that despite the flurry of activity in promoting public involvement, lay knowledge is marginalised and has limited impact on research decision-making. This article asks to what extent has power shifted from the scientific research community to the public? It discusses the meaning of power and models of public involvement and examines the development of public involvement in applied health research. It identifies public involvement in a range of decision-making: identifying priority areas for commissioning research; making decisions about which projects are funded; decisions about details of research design. Whilst there is evidence that the public voice is present in the composition of research proposals submitted to NIHR and in the decision-making about which projects are funded and how they are carried out, there is less evidence of a change in the power dynamic manifest in social relations between the scientific research community and the public. As a result the biomedical model remains dominant and largely unchallenged in research decision-making.

## Background

Public involvement in applied health research has been gathering pace in the UK. It has long been embedded in research funded by some charity sector organisations such as the MS Society. The National Institute of Health Research (NIHR), the leading funder of applied health research in the UK, has had public involvement in its principles from its inception in 2006. Public involvement is seen as key for the NIHR to achieve its mission of providing “a health research system in which the NHS supports outstanding individuals working in world-class facilities, conducting leading-edge research focused on the needs of patients and the public” [1]. An increased focus on public involvement is also discernible in other research funding agencies such as the UK Research Councils and the Wellcome Trust [2].

Although public involvement in research is by no means the exclusive domain of NIHR, they have undoubtedly channelled more resources than other organisations into its development and growth. It is claimed that NIHR has made more progress than the medical research charity sector in incorporating public involvement in their funding processes [3]. As the NIHR reaches its 10th anniversary it is timely to reflect upon the achievement. Few would deny that numerous partnerships have been established between funders and researchers on the one hand and members of the pubic and service users on the other and that the public now makes a contribution to the applied health and social care research portfolio. What is less clear is the extent to which the public is an equal partner in the process by which research is prioritised, funded, designed, conducted and disseminated. Whilst NIHR or other funding bodies rarely overtly address issues of power, the recent review of public involvement in NIHR talks of a future which brings together the public, researchers, health professionals, NHS staff and others as *equal partners* in creating knowledge” [4] p.9 (my emphasis). It is thus timely to ask, to what extent has there been a shift of power to the people.

## Theoretical perspectives

The phrase ‘power to the people’ was used as a slogan by the Black Panther movement in the US, as a title for a John Lennon song and as a catchphrase for 1970s UK sit-com character Citizen aka “Wolfie” Smith, a young Marxist ‘urban guerrilla’ in South London. Its use in this article does not refer to such calls for rebellion although it does view research through a political lens. It seeks to address the extent to which the rise of public involvement in applied health and social care research represents a shift of power from research funders and researchers to the voice of the people. Has public involvement in research changed the social relationship between researchers and those being researched?

Defining what is meant by ‘power’ is far from straightforward as it is a contested concept [5]. It has been defined as ‘power-to’ act to achieve some goal and ‘power over’ someone to make them do something that they would not otherwise have done [6]. Feminist theory has moved away from conceiving power as control and has reconceptualised power as a capacity or an ability, specifically, the capacity to empower [7]. The emphasis here is upon power as the capacity to transform and empower oneself and others. This underlies the ‘emancipatory’ research paradigm pioneered by the disability activist movement, which identifies reciprocity, gain and empowerment as key research priorities [8]. This in turn links to the notion of user-controlled research as research is unlikely to be emancipatory unless it is originated and directed by service users [9].

In the theory of power and community action, fewer voices have been more influential than the Brazilian educator Paolo Freire (1921–1997), in particular his concept of ‘conscientisation’ [10]. ‘Conscientisation’ is a process whereby critical thinking develops through dialogue and participation. At the beginning of this process people believe that they have no control over their lives and by the end of the transition they achieve empowerment defined as being able to think about and act on the conditions that shape their lives.

Arnstein’s ladder of involvement [11], which ascends from tokenism to full citizen control, follows the ‘conscientisation’ trajectory. It has been particularly influential in providing a conceptual framework for public involvement albeit with some criticism for being too static and too linear (see [12]). It has also been critiqued for focusing on power and not taking account of the unique contribution that public involvement knowledge and expertise brings to achieve a holistic understanding of contemporary health issues [13]. It has been argued that for this type of experiential knowledge to have full value, there must be a space in which both expert and lay knowledge can interact with each other on an equal basis [14]. This is the basis for the ‘citizen science’ movement which is based on the notion of establishment of equal partnerships between scientists and citizens [15]. The aim is that involvement of heterogeneous stakeholders with different motivations and values can challenge the fundamental approach adopted by scientific evaluation systems and promote new ways of formulating research questions, hypotheses, data collection, analysis and interpretation of the results [15]. However, in the UK at least, it has been argued that this equality of relationship between the public and professionals is not yet apparent. Indeed, Gibson et al. [14] suggest that public involvement is characterised by “systems which are controlled by salaried involvement professionals with committed volunteers being allocated a secondary, relatively impotent and subservient, role in organizational and bureaucratic labyrinths.” (p.534).

Beresford [16] provides a theoretical and critical perspective about different approaches to public involvement in research. He identifies consumerist and democratic models of involvement, the latter being more progressive. Both consumerist and democratic approaches are evident in user involvement in research [16]. The consumerist approach to user involvement is “framed mainly in market research terms of ‘improving the product’.” [16] p.97. It is consultative and in applied health research terms seeks feedback from the service user to enhance services. An example of an influential group originally based upon a consumerist approach is the National Cancer Research Institute’s (NCRI) Consumer Liaison Group which is now the NCRI Consumer Forum [17]. This was set up by the National Cancer Research Network and predates the NIHR. It currently has over 80 members who are involved in a range of NCRI activities “as experts in the experience of cancer” [17]. Whilst predominantly consultative, members are also involved in strategic decision-making and the Forum Chair is a lay position, both of which are more typical of a democratic approach. It is thus currently a crossover between consumerist and democratic approaches.

The democratic approach is “primarily concerned with people having more say in agencies, organisations, and institutions which impact upon them and being able to exert more control over their own lives.” [16] p.97. It draws upon the philosophy of human rights, participation, inclusion and autonomy. Whereas consumerist approaches are generally ‘top-down’ and managerial, i.e. initiated by the service system, the democratic approach is rooted in people's lives. The democratic approach is explicit about power, the distribution of power, collective action and empowerment such as that displayed in the activism from people living with HIV/AIDS demanding involvement in local and international public health policymaking (see [18]).

The notion of collective empowerment resonates with much of the rhetoric about public involvement in research. For example, a common mantra found on many NIHR websites is: “Public involvement in research is where our research is carried out ‘with’ or ‘by’ members of the public rather than ‘to’, ‘about’ or ‘for’ them.” [19]. The first bullet point in the terms of reference of INVOLVE, the body that drives public involvement in research for the NIHR, is “to promote the empowerment of the public to become more involved in research” [20]. But how effective has this empowerment been? Who is it empowering? Are the public empowered to drive the research agenda or at least are they on a pathway towards empowerment?

## Public involvement in applied health research

In the UK, there has been a fundamental shift in expectations about public involvement in applied health research. Going the Extra Mile, the report of a strategic review of public involvement in NIHR reported that: “The suggestion that members of the public are ‘subjects’ or ‘silent partners’ in research is no longer a tenable position to maintain for any research organisation wishing to fund high quality research. Partnership, reciprocity and openness are now fundamental to how research is done and to the successful translation of research results into practice” [4].

The NIHR has a clear expectation that there will be active involvement of members of the public in the research that it funds [4]. There are mandatory sections of the Standard Application Form for NIHR funding asking applicants to describe how they have involved the public and plan to do so if the research is funded as well as a requirement to write a clear plain English summary which can be understood and assessed by members of the public. In addition, the NIHR has encouraged the public to take part in research through, for example, its ‘OK to Ask’ campaign whereby members of the public and patients are encouraged to find out and take part in applied health research [21]. Public involvement in research has been given further impetus by the increasing emphasis on ‘Impact’ in the 2014 Research Excellence framework (REF) exercise, which is used to assess quality and allocate research funding to Higher Education Institutes in the UK. A total of 6975 impact case studies were submitted in REF 2014 to provide accountability for public investment in research and produce evidence of the benefits of this investment [22].

There is now a thriving and burgeoning public involvement infrastructure described by one contributor in the strategic review of public involvement across NIHR as, “a ‘frenzy of public involvement activity happening across the system” [4] p19. The national advisory group, INVOLVE, which was established in 1996, is now part of, and funded by, the NIHR. Its role is to support active public involvement in applied health and social care research by bringing “together expertise, insight and experience in the field of public involvement in research, with the aim of advancing it as an essential part of the process by which research is identified, prioritised, designed, conducted and disseminated” [23]. It organises workshops, a bi-annual conference and has a plethora of on-line resources about public involvement including an evidence library, research project database, publications of work it has led or commissioned, guidance about writing a plain English summary, briefing notes for researchers, examples about exploring the impact of public involvement, guidance on learning and development in the area, guidance and a cost calculator for payment to the public for various involvement activities and access to an advice service about benefits for members of the public who receive state benefits. INVOLVE also run the ‘People in Research’ website whereby researchers can seek members of the public to take part in their research and members of the public can find opportunities to get involved in research. There were 1.2 million hits on the INVOLVE website in 2014/15 [24].

A new strata of jobs with titles such as ‘Public Involvement Lead/Facilitator/Adviser/ Coordinator’ have emerged to deliver the public involvement in research agenda. Each of the many branches of the NIHR has designated public involvement posts including the research funding programmes, the clinical research networks, the research schools, the research centres and the clinical research facilities. There are estimated to be upward of 200 people with such titles working for NIHR [4], albeit many of these are part-time appointments. To give but one example, the NIHR Research Design Service, which supports researchers applying to NIHR programmes, in 2015 employed 31 people (totalling approximately 13 full-time equivalents) with a clear remit to develop and support public involvement in research.

Regional public involvement in research fora, such as People in Health West of England (PHWE), have been set up across most of England to pool expertise and share good practice. Further evidence of the number of researchers, research support workers and service users now involved in either facilitating, providing or engaging with public involvement in research is that over 500 people attended the bi-annual INVOLVE conference in 2014. The UK is seen as something of leader internationally in this field with one international contributor to the strategic review commenting that “if the UK is the adolescent in this area, we are the toddlers” [4] p20. Numerous articles have been written about various aspects of public involvement and journals such as ‘Research Involvement and Engagement’ published by BioMed Central have been established focusing on patient and public involvement and engagement in research.

In response to the call for applied health research to engage and involve members of the public, a growing number of service users and user groups have become involved. NIHR recently reported that in the last 5 years 1000 patients and members of the public were actively involved in directly supporting the NIHR infrastructure as reviewers, members of funding panels and other committees and groups [25]. However, this represents only a fraction of those involved in applied health research in the UK as it does not include the many independent patient groups, patients working with charities, nor the miriad of lay reviewers and advisers supporting applied health research activities not directly linked to NIHR. Those who become involved include individuals or groups who have experiential expertise of particular conditions and those who may not have direct experience of a condition or the area being researched but provide generic lay feedback. User groups have been set up across the country to facilitate the provision of feedback to researchers to ensure that their research meets the needs of the public. Furthermore, NIHR research networks have recruited Patient Research Ambassadors to help ensure that people using services are aware of opportunities for taking part in research [26]. A number of studies demonstrate that involvement in research is generally a positive experience for those patients and members of the public who take part [27, 28]. It may even be transformational in terms of redefining aspects of self in a more positive way [29].

There is a growing body of evidence that demonstrates the impact of public involvement in applied health research. The evidence base has been criticized in terms of scientific robustness as it draws heavily upon retrospective case studies [30]. However, robust evidence is now forthcoming from the NIHR funding call in 2010 which commissioned research into the impact of public involvement [31–33]. There are also at least three recently published systematic reviews [34–36]. There is also evidence that public involvement is positively associated with recruitment into studies on the NIHR Mental Health Research portfolio [37] and this was also reported in the RAPPORT study [33]. Other studies are developing tools to measure impact [38] or tools for devising public involvement impact assessment [39]. So what does this activity and evidence tell us about empowerment?

## To what extent has power shifted to the people?

The public voice has some representation at a strategic level within NIHR. The NIHR Advisory Board includes representatives from patient focused organisations and the NIHR Strategy Board, which advises on strategic issues relating to the management of the NIHR, includes both the Director of INVOLVE and the National Director for Patients and the Public in Research [40].

In terms of identifying priority areas for research, there are organisations that promote the public voice in agenda setting. For example, the James Lind Alliance Priority Setting Partnership [41], which is now part of the NIHR, brings patients, carers and clinicians together to identify and prioritise treatment uncertainties which they agree are the most important for research. Since 2007, these partnerships have identified the ‘Top 10’ research priorities in over 30 conditions ranging from acne to prostate cancer to palliative and end of life care [41].

There is some evidence of the impact of public involvement in setting priorities for asthma research [42]. However, there is less evidence about the extent to which the issues prioritised by the public lead to funded research projects in other areas. Most of the NIHR programmes fund commissioned research (topic specified by the programme) as well as having open calls (topic specified by the researcher providing it is within the remit of the programme). The majority of projects funded fall under this latter category, i.e. they are researcher-led. Furthermore, the James Lind Alliance is just one of the bodies that feeds into the process of identifying key research questions for funding (see [41]). The net result is that the majority of NIHR funded projects are either researcher-led or are on topics at the forefront of policy priorities, e.g. the themed call for proposals on dementia in 2011 was linked to UK’s then Prime Minister David Cameron’s high profile ‘dementia challenge’.

In terms of decision-making about what projects are funded, the NIHR funding panels now all include lay representatives who have direct input into these decisions. In addition, prior to panel meetings, research proposals are sent to both peer and lay reviewers. Nearly 700 public contributors were involved in reviewing over 1000 applications received by the NIHR in 2013/2014 [4]. There is thus clear evidence about involvement of the public in decision-making about which projects are funded. However, there remains concern about ‘tokenism’ and it is common to hear reports claiming that lay reviews are given less weight than peer-researcher/clinician reviews and also that the voice of public representatives on panels carries less influence than that of other members. There is though evidence to suggest that the influence of lay reviews and public representatives within funding panels has increased over the past 10 years [33]. Some of the participants in the RAPPORT study described this as a journey along a continuum of learning and understanding of public involvement, and one research funder detected “a sea-change” which was “possibly to do with…everything being reviewed by a public contributor” [33] p50.

There is a growing body of evidence confirming both depth and breadth of public involvement activity in the composition of research proposals submitted to the NIHR for funding [33]. It is now the norm for researchers to have dialogue with members of the public and service users when designing research and, to varying degrees, the public perspective is generally sustained once a project is funded. Lay reviewers and service users provide feedback to researchers applying to NIHR about the value of the research to the public and about how they perceive the design of the research will fare in real life health care settings. From a user perspective: is it measuring the key outcomes? Is it asking the right questions? Is it going to successfully recruit and engage potential research participants? How should the results be disseminated? This clearly has an impact on the design and conduct of funded research [43]. A review of ten case studies collected by the NIHR Research Design Service looking at the nature and impact of public involvement shows that it contributes to research proposals submitted for funding in a number of ways. This included: initiation of the research idea; feedback on relevance of research area; key aspects of the research design such as the inclusion of qualitative work; selection of outcome measures; advice on recruitment process and sample size calculation; advice about how to maintain adherence; advice on type or timing of intervention; reviewing the lay summary; development of research materials and project website [44].

There is also evidence that public involvement in non-commercially funded research is increasing. A comparison of responses in 2010 and 2012 to the public involvement question in the ethics applications to the National Research Ethics Service showed an increase in public involvement in non-commercial studies whereas for commercial studies there was little change in the scale of their involvement activities [3].

INVOLVE has collated a number of examples demonstrating sustained and wide-ranging impact across a range of applied health funded studies [45]. One of the studies they describe is ‘The RESPONDS Study. Bridging the knowledge and practice gap between domestic violence and child safeguarding: developing policy and training for general practice’, a research project funded by the Policy Research Programme [45]. The researchers set up survivor groups to provide advice and help to identity priority research topics. One issue to arise from these groups was concern about the impact of domestic violence on children and this was a key driver for the RESPONDS study. Once the RESPONDS project was funded the group continued to be involved. For example, they provided their views on the content of the GP training. One of the group members developed further research skills, conducting interviews with other survivors of domestic violence and co-authoring publications. The researchers on this project have clearly tried to break down barriers and empower this group of service users with some evidence of success. However, the lead researcher candidly reflected that, “Sometimes we overloaded the meetings with us talking too much. Even though the group has strong individuals who have no problems in speaking up, they have felt overloaded with information” [45] p.23. In response, they tried to make the meetings more interactive and establish an on-going relationship with the group members and to ensure that they understood their role in developing applications. This study is an example in which the researchers are clearly committed to public involvement, but nevertheless the research and the style of public involvement remains firmly led by the researchers.

The term co-production has emerged to provide a conceptual framework to equalise the relationship between professionals and service users. It has been championed in the field of social care (specifically the design and delivery of services) and the Social Care Institute for Excellence has produced a comprehensive guide [46]. Co-production has been defined in a number of ways but the one most pertinent to this paper is: “A relationship where professionals and citizens share power to plan and deliver support together, recognising that both have vital contributions to make in order to improve quality of life for people and communities”. (National Co-production Critical Friends Group cited in [46] p.6). The guide makes a clear distinction between co-production and participation with the former being defined by equality in the partnership between professional and service user and the latter being a form of consultation.

Likewise, the concept of ‘Experience-Based Co-Design’ has emerged in healthcare whereby staff, patients and carers work “together to identify improvement priorities, devising and implementing changes, and then jointly reflecting on their achievements” [47] p.11. In this model, the user voice has equal status [47].

The concepts of ‘co-production’ and ‘experience-based co-design’ are clearly applicable to public involvement in applied health research, specifically the relationship and balance of power between research funders/researchers on the one hand and service users/members of the public on the other. It maps onto the distinction noted earlier between consumerist and democratic approaches to involvement. The term co-production is becoming increasingly common to describe more democratic approaches to involvement whereas traditionally progressive approaches were associated with terms such as user-led, or user-controlled research, with a suggestion that research cannot be emancipatory unless it is user-led [48]. There is a subtle difference between the two as ‘co-production’ implies some equality of relationship whereas ‘user-controlled’ clearly locates the service user as dominant. The notion that anyone is’in control’ or’leading’ runs somewhat counter to a democratic model. Arguably, however, historically the user has had so little control in research, that there is a need for a model that clearly reverses this power imbalance. Furthermore, if the scientific community wants to involve and empower the public and is committed to incorporating their experiential expertise, it is natural that as part of this process service users will want to develop their own research agenda. This is the end point of the development of Freire’s ‘conscientisation’ (see [10]) discussed earlier in this paper, where people become empowered to act on the conditions that shape their lives.

There are examples of user-led and user-controlled research being carried out in the UK. Many of these are from the field of mental health such as those listed in the ‘Mental Health User/Survivor Research in the UK’ policy briefing [49]. There are organisations such as ‘Shaping Our Lives’ [50] which is a national organisation and network of user-led groups, service users and disabled people. It is user-led, committed to inclusive involvement, and undertakes research in consultation with service and users and represents their views. There are also examples of emancipatory research particularly in the field of disability studies [51].

However, examples of user-led approaches to research are less easy to find among research funded by NIHR and those that do exist are mainly in the domain of social care. In an NIHR INVOLVE sponsored report based on seven case studies of user-controlled research in health and social care [52], five of the selected cases were closely related to social care. The amount of funding they received was often very limited (with some under £10 k) and none were funded by NIHR (three were funded by charities, two by local authorities, one by a primary care trust and one by a statutory body). NIHR-funded user-led or user-controlled research is rare. For example, the NIHR Research Design Service in the East of England has supported 672 full submissions to peer-reviewed national funders from 2008/9-2014/15. Only a handful of these have been user-led and only one was funded.

A number of challenges and gaps have been identified with user-controlled research. User controlled projects often struggle to access funding and may encounter problems of credibility and discrimination [48], particularly as they often adopt methods at odds with positivist research values which underlie the paradigm of traditional biomedical research. In addition, a perennial problem is the difficulty in engaging with some groups of service users and the public. Older people and those from Black and minority ethnic (BME) groups are particularly underrepresented in user-controlled social care research despite the fact that older people are the main users of social care and people from BME groups are known to have unequal access [48].

Lack of representation from some groups is not only an issue in user-controlled research but has been noted among all forms and types of public involvement in research [4]. The public who volunteer to get involved in research are not representative of the general population with certain groups (e.g. youth, males, BME) being under-represented. There is also concern that the public representatives on funding panels are drawn from a small pool of people (often with a health or research background) and are not very representative of the general population [33]. There is evidence that as they become familiar with the working practices and conduct of panels and receive training they become ‘professionalised’ and adopt a professional rather than a lay discourse [53]. Whilst this may serve to enhance their credibility and influence on scientific committees, it inevitably involves a loss of ‘freshness’ and an increase in their alignment to the researcher view. A study of this process among people with experience of cancer who were involved with research panels noted that there were “no examples of individuals criticizing or challenging the dominant scientific model” [53] p.615.

Increasing diversity is a key recommendation in Going the Extra Mile [4]. It is important to widen representativeness of members of the public who become involved so that diverse ‘world views’ have a voice. There is a need for defined selection processes, limited terms of service and to consider methodologies to widen recruitment. Novel approaches are required as not all members of the public will feel comfortable in some settings such as sitting on a funding panel. A number of recent initiatives have emerged such as ‘People are messy: a new play about patient and public involvement in research’ ([54] targeted at young people aged 14+. The NIHR Collaboration for Leadership in Applied Health Research and Care (CLAHRC) in the East Midlands has established an East Midlands Centre for Black and Minority Ethnic Health and a Patient and Public Partners’ Council in an attempt to facilitate wider participation and involvement. Whilst such initiatives are welcome it is going to require a seismic shift to ensure that those members of the public with most power in the NIHR (e.g. those who sit on funding panels) are representative of the general population.

## Conclusions

The NIHR has succeeded in ensuring that patients and the public are a key part of the applied health and social care research infrastructure. There is evidence that the public voice is present in the composition of research proposals submitted to NIHR and in the decision-making about which projects are funded. There is also evidence that this can and does generally empower users who become actively involved in research [53]. There has not, however, been a concomitant transformation of the social relations of research, as envisaged by the emancipatory research movement. In spite of the fact that many if not most researchers now recognise the value of public involvement, the public voice is often not given the same weight as that of professionals. Accusations of tokenism and ‘box ticking’ continue, as well as the commonplace observation that the ‘usual suspects’ are being overused – i.e. that the same people from similar sectors of the population are selected to sit on research panels and steering groups. User-controlled research remains a side-lined activity which accounts for only a tiny proportion of funded health and social care research. This suggests that a consumerist approach is still predominant and that in reality the public voice has limited impact upon the research design or upon which research gets funded. The biomedical model would appear to dominate in research decision-making despite the participatory rhetoric.

However, it is important to acknowledge the value and benefits that have arisen from public involvement thus far. There are examples of co-production particularly in social care and mental health research and in organizations such as the NCRI Consumer Forum, patients and carers meet with professionals on equal terms and have equal voices. In addition, it is clear that many members of the public are comfortable with having a voice in a researcher-led model and, provided they are treated with dignity and respect, do not aspire to have a more influential role. Thus, it seems, that a variety of approaches can co-exist and a range of roles should be available to the public including research participant at one end of the spectrum and key decision-maker at the other. Whatever the model, the most important element of an effective partnership is a relationship framed by trust and mutual respect [34] in which everyone has a clear and well-defined role in the team, feels empowered to speak, listens respectfully and understands how and why decisions are made (see [33]).

Nevertheless, it is clear that the pendulum towards establishing more equal relationships, more democratic approaches and the process of ‘conscientisation’ still has a way to go. If public involvement is to value fully the user voice, it is important that these aspects are embedded in the landscape. Gibson et al. [14] have suggested an emancipatory framework to facilitate the creation of ‘knowledge spaces’ whereby the public and professionals can engage on equal terms. This requires the rejection of a knowledge hierarchy [14] and an acceptance that different forms of understanding are equally valid. An underlying respect for a plurality of views and opinions is the foundation of the concept of co-production. Providing trust is established, an equal relationship would enable different perspectives and different world views to challenge each other and the ensuing dialogue would enrich the research agenda. As Paolo Freire wrote, “One cannot expect positive results from an educational or political action program which fails to respect the particular view of the world held by the people. Such a program constitutes cultural invasion, good intentions notwithstanding.” [55] p.95). Whilst there are examples of empowerment among the public involved in research cited in this review, particularly in the fields of mental health, disability and social care, and in some cases this had been established for some time, it is vital that this is mainstreamed. The public must feel empowered to voice their views and challenge researchers if they are to be more than a hand-maiden to the established scientific community.
